# Complement Diagnostics: Concepts, Indications, and Practical Guidelines

**DOI:** 10.1155/2012/962702

**Published:** 2012-11-14

**Authors:** Bo Nilsson, Kristina Nilsson Ekdahl

**Affiliations:** ^1^Department of Immunology, Genetics and Pathology, Rudbeck Laboratory, Uppsala University, 751 85 Uppsala, Sweden; ^2^School of Natural Sciences, Linnæus University, 391 82 Kalmar, Sweden

## Abstract

Aberrations in the complement system have been shown to be direct or indirect pathophysiological mechanisms in a number of diseases and pathological conditions such as autoimmune disease, infections, cancer, allogeneic and xenogeneic transplantation, and inflammation. Complement analyses have been performed on these conditions in both prospective and retrospective studies and significant differences have been found between groups of patients, but in many diseases, it has not been possible to make predictions for individual patients because of the lack of sensitivity and specificity of many of the assays used. The basic indications for serological diagnostic complement analysis today may be divided into three major categories: (a) acquired and inherited complement deficiencies; (b) disorders with complement activation; (c) inherited and acquired C1INH deficiencies. Here, we summarize indications, techniques, and interpretations for basic complement analyses and present an algorithm, which we follow in our routine laboratory.

## 1. Introduction

The complement system is involved in numerous diseases and pathological conditions such as autoimmune disease, infections, cancer, allogeneic and xenogeneic transplantation, and inflammation [1]. The concentrations of various complement components and activation products have been measured in both prospective and retrospective studies of pathologic conditions, and significant differences have been found between groups of patients. However, in many diseases, it has not been possible to make predictions for individual patients because of the lack of sensitivity and specificity of many of the assays used. Basically, the indications for diagnostic complement analysis today can be divided into three major categories: (a) acquired and inherited complement deficiencies; (b) disorders with complement activation; (c) inherited and acquired C1INH deficiencies. Here, we give a personal view of how we perform basic complement investigations in our routine diagnostic laboratory.

## 2. The Complement System

### 2.1. Complement System Physiology

The complement system has a primary function in host defense and clears the body of foreign cells, microorganisms, and cell debris, either by direct lysis or by recruitment of leukocytes that promote phagocytosis and cytotoxicity (recently reviewed in [2]). It consists of more than 40 plasma and cellular proteins (receptors and regulators). The central complement reaction is the cleavage of C3 into C3b and C3a, which is promoted by two multimolecular enzyme complexes, the C3 convertases, which are assembled by three different recognition and activation pathways. The classical pathway (CP) is triggered by the formation of antigen-antibody complexes (immune complexes), which bind the C1 complex (C1q, C1r_2_, C1s_2_), and the lectin pathway (LP) by the binding of mannan-binding lectin (MBL) or ficolins to carbohydrates and other pathogen-associated molecular patterns. Both these events lead to the assembly of the CP/LP C3 convertase, C4b2a. The alternative pathway (AP) may be triggered directly by foreign surfaces, for example, by microorganisms or man-made biomaterials, which do not provide adequate downregulation of the AP C3 convertase, C3bBb. This convertase is stabilized by properdin, which also recently has been reported to act as a recognition molecule that is able to form a nucleus for convertase assembly [3].

**Figure 1:**
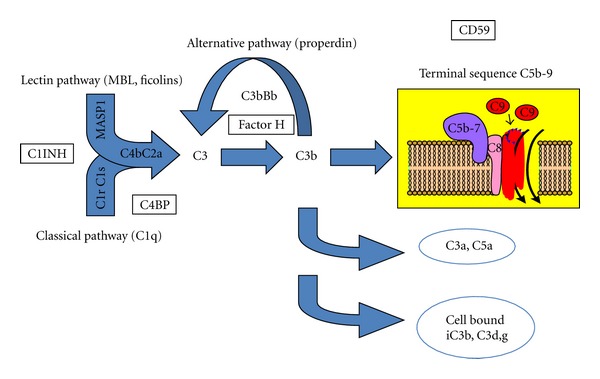
Overview of the complement system. Recognition by the lectin and classical pathways leads to the assembly of the C4b2a convertase, which cleaves C3. This reaction is greatly amplified by the alternative pathway, generating more C3b, and ultimately initiating the terminal sequence. The fluid phase anaphylatoxins, C3a and C5a, together with the cell-bound opsonins iC3b and C3d,g, facilitate phagocytosis. The main inhibitor of each step in the cascade is indicated in boxes: C1INH for initiation, C4BP for the classical pathway, factor H for the alternative pathway, and CD59 for the terminal sequence.

The nascent C3b molecule has the specific property of binding to proteins and carbohydrates via free hydroxyl or amino groups, resulting in covalent ester and amide bonds, respectively. The AP serves as a major amplification loop, so an initial weak stimulus mediated by any of the pathways may be markedly enhanced. The activation pathways converge in a common pathway to form the membrane attack complex (C5b-9), which elicits cell lysis by insertion itself into the lipid bilayer of cell membranes. The anaphylatoxins C3a and C5a activate and recruit leukocytes, while target-bound C3 fragments (C3b, iC3b, C3d,g) facilitate binding to and activation of the recruited cells (Figure 1).

### 2.2. Complement System Regulation

*In vivo*, the complement system is controlled by multiple soluble and membrane-bound regulators [2]. Most of the regulators are members of the “regulators of complement activation” (RCA) superfamily, which mainly regulate the convertases. The plasma proteins factor H and C4b-binding protein (C4BP), the membrane proteins complement receptor 1 CR1 (CD35), decay acceleration factor, DAF (CD55), and membrane cofactor protein MCP (CD46) all belong to this family and exert their action by functioning as cofactors for plasma protease factor I and/or by accelerating the decay of the convertases. In addition, CD59 is a regulator of the C5b-9 complex, and C1 inhibitor (C1INH) regulates the proteases of the C1 complex (C1r and C1s) and MASP-1, -2, and -3 of the LP (Figure 1).

### 2.3. Complement System Pathology

Excessive complement activation is part of the pathogenesis of a large number of inflammatory diseases. The pathologic effect may be due either to an increased and persistent activation, for example, caused by the presence of immune complexes (such as in systemic lupus erythematosus, SLE, and related disorders), or to a decreased expression or function of various complement inhibitors (see examples below), or to a combination of the two, as discussed in [4] and quoted references.

Ischemia, followed by reperfusion of an organ or blood vessel, occurs in a number of conditions, such as during heart infarction or stroke. It can also occur during medical treatment modalities such as cardiovascular surgery facilitated by cardiopulmonary bypass as well as after transplantation, in both allogeneic and xenogeneic settings, and can often be accompanied by ischemia/reperfusion (IR) injury. Complement activation and insufficient regulation play important roles in IR injury, and activation by all three pathways of complement has been implicated in the damage. The result is an multifunctional inflammatory process, involving generation of anaphylatoxins, upregulation of adhesion proteins and tissue factor on endothelial cells, and recruitment and extravasation of PMNs, as summarized in [5] and cited references.

The net result of this dysregulation between initiators and inhibitors of complement activation in all these diverse conditions is a prolonged complement activation, which ultimately results in tissue damage.

## 3. Examples of Indications for Complement Analysis

### 3.1. Inherited and Acquired Complement Component Deficiency

#### 3.1.1. Complement Factor Deficiencies (General)

Complement deficiencies are associated with an increased risk of infections and, in some cases, autoimmunity [6]. A deficiency of one component within the CP (the subunits of the C1 complex, C2, and C4) not only predisposes an individual to infections but also to immune-complex diseases, that is, SLE-like conditions; deficiencies in other components are mainly associated with infections [4]. The point in the complement cascade at which a deficiency occurs determines the specificity of the infection (in almost all cases bacterial) that affects a patient, the most common being caused by *Neisseria*, *Haemophilus*, and *Pneumococci* [7]. A deficiency of MBL in the lectin pathway increases the risk of any type of infection: bacterial, viral, or fungal, particularly in various immunosuppressive states, such as during the neonatal period, or during immunosuppressive treatments [6].

#### 3.1.2. Monitoring of Complement Regulatory Drugs

Eculizumab is the first approved complement inhibitor in clinical use. It is a humanized monoclonal antibody that binds to complement component C5, hindering its proteolytic activation, and thereby inhibiting the generation of the anaphylatoxin C5a as well as the initiation of the lytic C5b-9 complex. The main indication for this complement inhibitor is for the treatment of paroxysmal nocturnal hemoglobinuria, PNH [8], and atypical hemolytic uremic syndrome, aHUS [9], for which it has orphan drug designation. In addition, successful off-label use has been reported in shiga toxin-induced hemolytic uremic syndrome [10] and refractive membranoproliferative glomerulonephritis, MPGN [11], as well as for reversing ongoing rejection in ABO-incompatible transplantation [12]. The functional effect of this treatment, and of other future complement inhibitors, *in vivo* may be monitored by complement analysis (see below) [12].

#### 3.1.3. Paroxysmal Nocturnal Hemoglobinuria (PNH)

Paroxysmal nocturnal hemoglobinuria (PNH) is a rare hematologic disorder in which the afflicted patients suffer from hemolysis with acute exacerbations that lead to anemia, as well as from an increased risk of venous thrombosis. The disease is caused by acquired somatic mutations of the X-chromosomal gene *PIG-A* in a limited number of hematopoietic stem/progenitor cells [13]. The afflicted cells lack the enzyme encoded by *PIG-A*, which is essential for synthesizing the GPI (glycosylphosphatidylinositol) anchor; these cells (leukocytes, erythrocyte, or platelets) are therefore unable to bind GPI-anchored proteins, including the complement regulators DAF and CD59. Consequently, they are susceptible to attack by autologous complement ([14] and references therein). PNH will not be further mentioned in the text but the current state of the art regarding the diagnosis and management of PNH is described in detail in [15].

### 3.2. Disorders with Complement Activation

#### 3.2.1. SLE and Urticarial Vasculitides

SLE and urticarial vasculitides belong to the group of autoimmune immune complex diseases [16, 17]. Cryoglobulinemia, rheumatoid arthritis with vasculitis, and rare cases of Wegner's and Henoch Schönlein disease also belong to this group [18, 19]. Complement analyses (see below), most notably the assessment of CP function, and the concentration of individual complement components, for example, C1q, C4, and C3 can be used for differential diagnosis and to follow disease activity. Detection of autoantibodies against C1q and C3 may corroborate diagnosis [20–22]. Hypocomplementemic urticarial vasculitis syndrome (HUVS) presents with severe complement consumption via the CP and anti-C1q antibodies [17].

#### 3.2.2. Membranoproliferative Glomerulonephritis

Membranoproliferative glomerulonephritis (MPGN) types II are associated with C3 nephritic factors (C3Nef) [23]. C3Nef is an autoantibody directed against a convertase. In MPGN type II, the antibody is directed against the AP convertase, resulting in a dramatically extended half-life of the convertase. The consequence of this antibody association is a profound C3 consumption, leading to a functional deficiency of C3. The C3 consumption is accompanied by the generation of C3d,g, indicating a prominent activation of C3. In some cases of C3Nef (type II), the stabilized convertase also cleaves C5. Detection of C3Nef supports the diagnosis of MPGN. The resulting severe C3 deficiency may theoretically increase the risk of bacterial infections.

#### 3.2.3. Poststreptococcal Glomerulonephritis (PSGN)

In individuals suffering from poststreptococcal glomerulonephritis (PSGN), C3 and C5 may be consumed and sC5b-9 generated, in the rehabilitation period, up to 6 months after the infection [23]. The levels may be very low and, as in the case of C3Nef, there is a theoretical risk of other bacterial infections. C3d,g levels are elevated and, in particular, the ratio C3d,g/C3 is high. The mechanism of C3 consumption is not known, but the major difference in complement activation compared to C3Nef is that PSGN is associated with a concomitant consumption of properdin [23].

#### 3.2.4. Atypical Hemolytic Uremic Syndrome (aHUS)

Atypical hemolytic uremic syndrome (aHUS) is a disease that appears in the childhood and is characterized by microangiopathic hemolytic anemia, thrombocytopenia, and acute renal failure resulting from membranoproliferative glomerulonephritis. The cause of this disease is dysregulated complement activation following a mutation in factor H, factor I, MCP, or factor H-related proteins (FHR) 1, 3, or 5, that impairs the functioning of these inhibitors. In addition, mutations in C3 and factor B that lead to dysregulated activation have also been described [24] and referenced therein [25].

The cells that are affected are erythrocytes, platelets, and endothelial cells, including those of the mesangium of the kidney. Mutations within the factor H gene are the most common cause of aHUS. The majority of these mutations are localized in the C-terminally located short consensus repeats (SCRs) 19-20, which are involved in the binding of factor H to the cell surface. Factor H binds to carbohydrates, and heparan sulfate and sialic acid are common ligands for the protein. As in the case of factor H deficiency, engagement of the AP leading to consumption of C3 and generation of C3d,g (or other C3 fragments) and C5b-9 may be seen. In rare cases, the mobility in SDS/PAGE followed by western blotting may differ from that of normal factor H. Patients with suspected aHUS should be handled by laboratories specialized in determining mutations in all activators and soluble and cell bound regulators of the AP.

### 3.3. Inherited and Acquired C1INH Deficiency

Hereditary angioedema (HAE) and acquired angioedema (AEE) are rare disorders that are caused by a C1INH deficiency and, in rare cases, by mutations of the contact system proteins [26]. These diseases are caused by an unregulated formation of bradykinin of the contact system, and hence they are not primarily diseases of the complement system. However, the diagnosis is based on complement analyses. HAE is subdivided into three types (I–III), which can be identified only by laboratory analysis. The type I form of HAE is characterized by a low concentration and function of C1INH, and type II by a normal concentration of a dysfunctional C1INH. The third form, type III, which is not due to low C1INH function, is in many cases estrogen dependent. This is a heterogeneous group, which is less well characterized, than the other two forms. Some of the patients with type III have mutations in the contact system *F12* gene, coding forms of FXII with gain of function [26].

Acquired deficiencies of C1INH may occur in lymphoproliferative and autoimmune diseases, due to formation of paraproteins, for example, M-components and autoantibodies against C1INH, respectively, which result in consumption of the protein [27, 28].

## 4. Analytical Methods

Available complement assays have recently been comprehensively reviewed [1]. Here, we present analytical methods, which are suitable for routine diagnostics.

### 4.1. Quantification of Individual Complement Components

**Figure 2:**
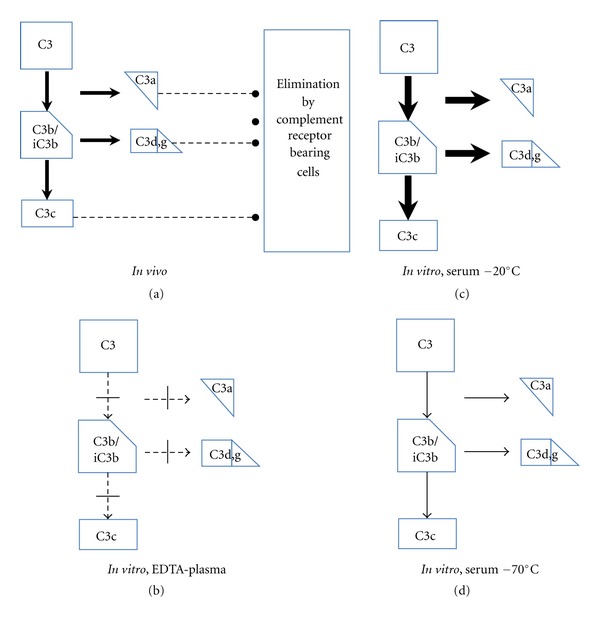
Activation and consumption of complement *in vivo* and *in vitro*. *In vivo*, complement is activated, and C3 gives rise to the activation products C3a, C3b/iC3b, C3d,g, and C3c (indicated by arrows). *In vivo*, a fraction of these complement products are bound to and eliminated by different complement receptor-bearing cells in contact with plasma (a). When blood is drawn in the presence of EDTA, all further complement activation is inhibited (b). The complement system is active in serum and may be activated to a substantial degree *in vitro* in maltreated samples (c), but it can be kept essentially intact in properly handled samples (d). The thickness of the arrows in each panel indicates the degree of C3 cleavage.

The concentration of individual proteins is determined by various types of immunoassays. The most common approach in clinical practice is to use immunoprecipitation assays, today mainly nephelometry and turbidimetry. In the latter techniques, polyclonal antibodies against the protein of choice (e.g., C1q, C1INH, C4, C3, or factor B) are added to the sample, forming complexes that will distort a detecting light beam that is passed through the sample. These techniques, which use polyclonal antibodies to detect the total amount of the antigen in question, are relatively robust with regard to the effects of suboptimal sample handling, such as proteolytic cleavage or denaturation of the target proteins. For example, the polyclonal antibodies raised against C3c used in such assays will recognize C3c-fragment containing intact, nonactivated C3 as well as its inactive proteolytic fragments C3b, iC3b, and C3c, on an equimolar basis. Similarly, anti-C4c antibodies will detect the corresponding forms of C4. If the sample is poorly treated, resulting in fragmentation of the intact protein, determination of the c-fragment (C3c or C4c) ensures that the determined concentration is similar to that *in vivo* (Figures 2(a) and 2(c)). However, this assay gives no information about the conformation or activation state of the protein and it is used mainly to determine the protein's *in vivo* concentration (i.e, to monitor consumption) (see Figure 2). Consequently, to give a measure of complement activation *in vivo*, it is necessary to measure an activation fragment/product, for example, C3d,g (Section 4.2).

### 4.2. Quantification of Activation Products

A number of complement proteins are activated and inactivated by sequential proteolytic cleavages that are accompanied by conformational alterations. These reactions have been studied most extensively for C3. Therefore, the strategy used to demonstrate that complement activation has occurred *in vivo* relies on detecting complement activation products (with altered size and conformation or composition) in the sample. Activation of C3 may be monitored either by identifying the protein fragment C3a, which is generated in the first proteolytic cleavage step, or the C3d,g fragment, which is the end product (together with C3c) (Figure 2). These peptides vary greatly with regard to their half-lives (*T*_1/2_) *in vivo*: approximately 0.5 hr for C3a [29] and 4 hr for C3d,g [30]. In addition, there is a great risk that C3a will be generated *in vitro* during improper handling of samples. Consequently, since C3d,g is a more robust marker, it is more suitable for diagnostic use, while the generation of C3a is more suitable for *in vitro* analysis in experimental settings.

Complement activation gives rise to products with different properties than those of the zymogen molecules. Therefore, assays for the determination of complement activation products generally work according to one of two principles: either (1) the zymogen molecules and products are fractionated according to size before being detected by polyclonal antibodies, as described above for C3d,g (below), or (2) monoclonal antibodies specific for amino acid sequences that are hidden in the native zymogen molecule but exposed upon activation (so-called neoepitopes) are used. Since only the activation product and not the zymogen molecule will be detected in this assay, it is not necessary to include a precipitation step. Most available assays for C3a, C3b/iC3b/C3c, and C5b-9 (below) are based on neoepitope monoclonal antibodies [31–33].

C3d,g is detected by nephelometry/turbidimetry or enzyme immunoassays (EIA) using polyclonal antibodies (see Section 4.1). However, since these antibodies also recognize intact C3, C3b, and iC3b in addition to C3d,g, it is necessary to remove these larger molecules by polyethylene glycol (PEG) precipitation prior to analysis. C3d,g is continuously generated *in vivo* under normal conditions, presumably as part of the physiological turnover of C3. Therefore, it is useful to determine the ratio between the C3d,g level and the total level of C3 in order to monitor ongoing complement activation (C3d,g/C3), for example, during a flare in SLE [34].

The final step in the complement cascade is the formation of the C5b-9 complex, which is inserted into cell membranes, thereby causing cell damage and/or lysis. sC5b-9, the soluble form of this high molecular weight complex, can be quantified in the fluid phase as a marker of full complement activation, by using an EIA with a monoclonal antibody specific for a neoepitope in C9, which is exposed in complex-bound but not intact C9. Detection of the formed complexes is performed by using polyclonal antibodies against C5 or C6 (i.e., another protein present in the same macromolecular complex) [33].

Since all these activation markers can be rapidly produced by complement activation *in vitro*, these assays are sensitive to preanalytical factors, so it is of critical importance that the samples are collected and handled properly (see Section 4.5).

### 4.3. Quantification of Complement Function

The function of each of the complement activation pathways is dependent on the integrity of each of the participating components, and therefore a deficiency in a single component will affect the activity of the whole cascade. One major advantage of functional tests that monitor a whole activation pathway from initiation to the effector phase (lysis) is that they will detect both deficiencies in complement components and consumption-related decrease of complement activity, thereby combining information obtained using the types of assays described above.

Complement activation by the CP is studied in hemolytic assays utilizing sheep erythrocytes coated with rabbit antibodies (IgM with or without IgG). When serum is added, the C1 complex will bind, leading to formation of the CP convertase, which activates C3. Activation of C3 then initiates the assembly of the C5b-9 complex, which ultimately results in erythrocyte lysis [35, 36].

Complement activation by the AP is studied by using rabbit or guinea pig erythrocytes, which are spontaneous activators of the human AP. When the cells are incubated in serum with the addition of EGTA to chelate Ca^2+^ (to inhibit activation by the CP and LP), the AP convertase is formed, resulting in the activation of C3 and subsequent lysis of the erythrocytes [36, 37].

Hemolytic assays can be performed in different ways; the original assays, the so-called CH_50_ and AH_50_, are based on titration of the amount of serum necessary to lyse 50% of specified amount of cells [35, 37]. The considerably less laborious and faster one-tube assays, which only necessitate the use of one serum concentration, give corresponding results [36]. The commonly used hemolysis in gel technique is performed with erythrocytes cast in an agarose gel. The serum is added to punched holes and diffuses into the gel whereby the erythrocytes are lysed. This technique is excellent for screening for complement deficiencies but does not provide a quantitative measurement [38].

More recently, a method comprising three separate EIAs which for the first time enables the simultaneous determination of all three activation pathways (including the LP) has been reported. The assay can best be described as a solid-phase functional test, since it comprises recognition molecules specific for each pathway (IgM for the CP, mannan or acetylated bovine serum albumin, BSA, for the LP, and LPS for the AP). These molecules are coated onto ELISA plate wells, and then serum is incubated under conditions in which only one pathway is operative at a given time and the other two pathways are blocked. The final step in each EIA is the detection of the resulting C5b-9 complex by a monoclonal antibody against a neoepitope in complex-bound C9 [39]. This assay is commercially available (Wielisa, Wieslab, Lund, Sweden). The correlation between this assay and conventional hemolytic assays is linear for the CP and for the AP at high activity but not at lower levels.

**Figure 3:**
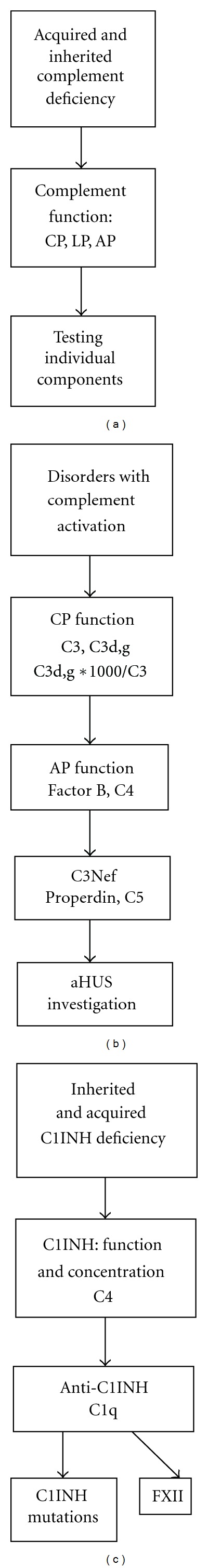
Algorithm for complement analyses. The aims are to diagnose complement deficiency in patients with recurrent bacterial infections (a), diagnose the cause of their persistent complement activation (b), and to dissect the cause of C1INH deficiency (c). See text (Section 5.1) for details.

These functional techniques are particularly useful for (1) identifying congenital deficiencies and (2) monitoring fluctuations in complement function, for example, in SLE patients during exacerbations. A tentatively identified deficiency can be confirmed by concentration determination using a protein-specific assay and by experiments in which the patient sample is reconstituted with the relevant protein. (Most plasma complement components are commercially available). These analyses will provide information whether it is a functional deficiency or a total lack of the protein (Figure 3(a)).

### 4.4. Quantification of Autoantibodies to Complement Components

C3Nef are autoantibodies that bind to components of the AP convertase, thereby prolonging its functional *T*_1/2_ and leading to increased complement activation. There are two basic assays for the detection of C3Nef: an AP-dependent hemolytic assay employing noncoated sheep erythrocytes [40] (in contrast to the CP hemolytic assay described above) and an assay to assess fluid-phase C3 cleavage, detected by, for example, crossed immunoelectrophoresis [41]. C3Nef are designated as C3Nef type I or II, based on the pattern of reactivity in these two assays. Over all, C3Nef type I predominately stabilizes the C3 AP convertase, while C3Nef type II also results in C5 cleavage [42].

Recent efforts to improve detection of C3Nef include construction of ELISA-based functional assays using nickel-stabilized C3bBb, and real-time monitoring of the formation and decay of C3-convertase formation using surface plasmon resonance, SPR [43, 44].

Anti-C1q autoantibodies are found in several autoimmune conditions and also in healthy controls. The assay is performed as follows: coating ELISA plates with purified C1q, incubation of patient serum and binding of true anti-C1q autoantibodies to the collagen part of C1q, and detection of bound IgG antibodies using antihuman IgG antibodies. In order to avoid that IgG-containing immune complexes in the samples bind to the coated C1q it is necessary to perform the assay in the presence of high concentrations of NaCl, typically 0.5–1.0 M, which dissociates the binding of C1q to IgG-containing immune complexes. The role of anti-C1q autoantibodies and methodological considerations are discussed in detail in [22].

Immunoconglutinins (IKs) are autoantibodies against fragments of C3 or C4 that affect the functioning of these components and are found in inflammatory states and autoimmune diseases, including SLE. IKs can be detected by EIA, using C3-coated wells for capture and polyclonal antihuman IgG, IgA, or IgM antibodies for detection [20, 21, 45].

### 4.5. Collection and Storage of Samples

EDTA is the only anticoagulant that completely inhibits any complement activation *ex vivo*, and EDTA-plasma should be used for the quantification of complement components and their activation products. Heparin and citrate are insufficient inhibitors of complement activation and are thus unsuitable anticoagulants for complement analysis. Serum or plasma anticoagulated with a specific thrombin inhibitor, such as lepirudin, is used for the quantification of complement function and autoantibodies. Alternatively, EDTA-plasma can be used for the functional assays, provided that the samples are transferred to Veronal buffer with Ca^2+^ and Mg^2+^ (to enable complement activation) and lepirudin (to inhibit coagulation) [34]. Plasma and serum should be separated within 2 hr of collection and frozen at −70°C. Storage at −20°C should be avoided. If samples need to be transported, they should be sent in packages containing dry ice (with samples pre-frozen at −70°C). Prior to analysis, the samples should be thawed rapidly, preferably in a 37°C water bath, and then kept on ice.

## 5. Concept and Interpretations

### 5.1. Algorithm for Complement Analyses

In this section we summarize the indications for complement analyses and present an algorithm, which we follow in our laboratory when we perform complement diagnostics. The algorithm is shown in Figure 3. Complement analysis is generally undertaken for three different indications.

**(A) Acquired and Inherited Complement Deficiency**

Complement deficiencies may be due to treatment with complement-inhibiting drugs such as the newly introduced eculizumab. Quantitative functional assays can be used to test the effect of the drug *in vivo*. This indication will probably increase with the introduction of new complement regulatory drugs and may also include, for example, measurement of sC5b-9 to monitor administration of eculizumab for the different indications mentioned in Section 3.1.2. Another important indication for complement analysis is recurrent severe invasive bacterial infections that may be due to an inherited complement deficiency. The first step in ascertaining an inherited complement deficiency (Figure 3(a)) is to determine complement function by all three pathways, either by hemolytic tests or by Wielisa. No hemolytic test has been described for the LP. Therefore, CP and AP hemolytic assays may be complemented with at least determination of the concentration of MBL as a surrogate marker for LP function. If a defect is found, it can be verified by a specific concentration assay for the lacking component, and by reconstituting the functional assay with the specific deficient protein. If relevant, genetic analysis may be performed.

**(B) Disorders with Complement Activation**

The initial step in our algorithm to determine and assess the cause of complement activation (Figure 3(b)) is to determine CP function and the concentrations of C3 and C3d,g. Computer analyses have shown that if these three parameters are within the reference values, the sample is normal with regard to complement activation, with 95% certainty (Nilsson, UR and Groth, T, unpublished data). Samples with values outside the reference intervals are then tested for AP function and determination of factor B and C4 concentrations. Combined, all these assays represent functional tests and markers for the CP, AP, and terminal pathways (Figure 1). If no plausible explanation for a solitary high C3d,g/C3 ratio is found and if the clinical condition necessitates further investigation, the next step is to determine the presence of C3Nef and the concentrations of properdin and C5. Cases where aHUS is suspected should be transferred to a laboratory specialized in aHUS diagnostics.

**(C) Inherited and Acquired C1INH Deficiency**

The cause of a C1INH deficiency (Figure 3(c)) is dissected by analyzing the concentration and function of C1INH and the concentration of C4. In type I deficiencies both the C1INH concentration and the function are low. In type II only the function is deficient. In both deficiencies, C4 is often low as a result of remaining C1s activity. Samples, in which an acquired deficiency is suspected, are analyzed to determine the concentration of C1q and the presence of autoantibodies against C1INH. In acquired C1INH deficiency due to lymphoproliferative disease, paraproteins may lead to C1q consumption and in autoimmune rheumatic disease there may be anti-C1INH antibodies. The final step in the investigation of HAE includes determination of mutations in C1INH by specialized laboratories. If no aberrations are found in C1INH function despite clinically typical angioedema, then analyses of factor XII function and genetic determination may be performed. The current state of the art regarding the diagnosis and management of HAE and AAE is described in detail in [46, 47].

### 5.2. Differentiation between *In Vivo* and *Ex Vivo* Activation

Activation of the complement system both *in vivo* and *in vitro* leads to the generation of activation products, for example, the anaphylatoxins C3a and C5a and the fragments of C3 and C4 produced by sequential proteolysis, as described in Section 2.1 above. *In vivo*, most of these products interact with receptors on various cells in contact with plasma (erythrocytes, leukocytes, endothelial cells, and fixed macrophages, etc.) and are rapidly cleared from the circulation, leading to consumption of the components (Figure 2(a)). In contrast, complement activation *ex vivo*, in the collected serum or plasma samples, will generate the same products, but in this situation there are no cells present, so the activation products will remain in solution (Figures 2(b), 2(c), and 2(d)). Functional assays will in both cases be low but only samples where activation has occurred *in vivo* will show consumption of individual complement components.

### 5.3. Interpretation of Laboratory Results, with SLE as an Example

**Figure 4:**
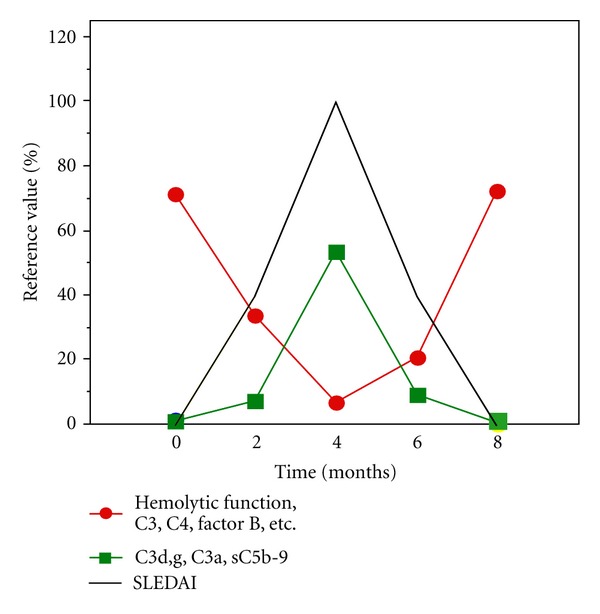
Complement activation and hemolytic function during an SLE exacerbation. C3, C4, factor B, and other components are consumed, leading to a depression in hemolytic function (red line). The resulting activation products, C3d,g, C3a, and sC5b-9 (green line), peak concomitantly with the SLE disease index (SLEDAI).

Use of the commonly employed combination of C3 and C4 concentrations to monitor complement in immune complex disease should be avoided, since both the sensitivity and specificity of these measurements are low. For example, SLE patients may have inherently low concentrations of C4 as a result of a low gene copy number [48]. Immune complex diseases are characterized by a moderate-to-severe CP activation and consumption. This activation reflects the activity of the disease, and many times the complement consumption precedes an exacerbation of the disease. In order to determine the first and initial complement status of the patient, a functional assay (e.g., hemolytic or EIA-based), combined with an assay to determine complement activation products (e.g., C3d,g, C5b-9), is necessary. By using this combination of tests, the laboratory can determine whether a low complement function measured by the functional test is really the result of a consumption/activation or is caused by a deficiency/dysfunction. For monitoring of the patient, a single test can be used. For SLE, the C1q concentration or a hemolytic assay, such as the single-tube CP assay or CH_50_, can be used. Certain cryoglobulinemias can easily be detected by functional complement assays. These patients may consume complement via the CP already *in vivo*, but this activation is often amplified *in vitro* as a result of the handing of the sample, leading to a very low CP function (if serum is used) without a corresponding consumption of CP components, as determined by immunochemical assays (if EDTA is used). These samples may be misdiagnosed as deficiencies (Figure 4).

### 5.4. Interpretation of Laboratory Results, General

As an illustration of what was discussed in the previous section we have constructed Table 1. The first patient is an SLE with complement activation triggered by the CP. This patient has consumed components *in vivo* via the classical and terminal pathways. Thus, the function via CP is low. We also see that there is consumption of C4, C3 but not of factor B. There is also generation of C3d,g and the ratio C3d,g/C3 is above the reference value. In the second SLE patient, the function via CP is also low but there is no sign of consumption of complement components or C3d,g generation. This patient has a C2 deficiency. This illustrates that assessment of complement function in order to detect complement deficiencies is not sufficient without further analyses of complement consumption/activation. Another confusing condition is cryoglobulinemia, which will present an identical profile as in the C2 deficient patient, mimicking a deficiency. Here, all determinations of individual components and of complement fragments are measured on the EDTA-plasma in which no further complement activation occurs after withdrawal of blood. By contrast, the serum sample may be activated by cryoglobulins (immune complexes) and therefore the CP function will be affected. Such mistakes can be avoided if the samples are drawn, centrifuged at 37°C, and CP function is analyzed without freezing of the sample or if the CP function is analyzed on the EDTA-plasma (see above).

## 6. Conclusions

In summary, complement analyses for individual patients are useful in a relatively limited number of conditions, which are summarized in Table 2. The profile for the typical case of each condition is presented in the Table. Indications for complement analyses will increase with introduction of new regulatory drugs of complement and with new assays for example, it is likely that assays of the LP will generate new indication for complement investigations.

## Figures and Tables

**Table 1:** Complement function of the CP and AP, plasma concentrations of C3, C4, factor B, and C3d,g, and the C3d,g∗1000/C3 ratio in one patient with SLE and one patient with a C2 deficiency.

	CP (%)	AP (%)	C3 (g/L)	C4 (g/L)	Factor B (g/L)	C3d,g (mg/L)	C3d,g∗1000/C3
SLE	15	45	0.53	0.07	0.18	7.0	13.2
C2 def	5	97	0.80	0.14	0.30	3.5	4.4
Reference interval	80–120	50–150	0.67–1.29	0.13–0.32	0.16–0.44	<5.3	<5.3

**Table 2:** Complement pathology, differential diagnostics.

Analyses	HAE/AAE	SLE, urticarial vasculitis	PSGN	MPGN II and III	Complement deficiencies
CP	N	L	L	L	*
AP	N	N (L)	L	L	*
C1INH (conc)	L	N	N	N	*
C1INH (funct)	L	N	N	N	*
C1q	(L)	L	N	N	*
C4	L	L	N	N	*
C3	N	L	L	L	*
C5	N	—	L	(L)	*
Factor B	N	N	L	N	*
Properdin	N	—	L	N	*
C3d	N	H	H	H	N
C3d,g/C3	N	H	H	H	N

*Variable (dependent on which component is defective); N: normal; L: low; H: high.

## Abbreviations

AEE: Acquired angioedema
aHUS: Atypical hemolytic uremic syndrome
AP: Alternative pathway of complement
BSA: Bovine serum albumin
C1INH: C1 inhibitor
C3Nef: C3 nephritic factor
C4BP: C4b-binding protein
CP: Classical pathway of complement
CR1: Complement receptor 1 (CD35)
DAF: Decay acceleration factor (CD55)
EIA: Enzyme immunoassay
FHR: Factor H-related protein
GPI: Glycosylphosphatidylinositol
HAE: Hereditary angioedema
HUVS: Hypocomplementemic urticarial vasculitis syndrome
IKs: Immunoconglutinins
IR: Ischemia/reperfusion
LP: Lectin pathway of complement
LPS: Lipopolysaccharide
MBL: Mannan-binding lectin
MCP: Membrane cofactor protein (CD46)
MPGN: Membranoproliferative glomerulonephritis
PEG: Polyethylene glycol
PNH: paroxysmal nocturnal hemoglobinuria
PSGN: poststreptococcal glomerulonephritis
RCA: regulator of complement activation
SLE: systemic lupus erythematosus
SLEDAI: SLE disease index
*T*_1/2_: half-life.
